# 1-Methyl-5-nitro-1*H*-imidazole

**DOI:** 10.1107/S1600536811041705

**Published:** 2011-10-29

**Authors:** Ying Diao, Wen-Yan Wang, Zhi-Hua Wei, Jian-Long Wang

**Affiliations:** a School of Chemical Engineering and Environment, North University of China, Taiyuan, People’s Republic of China

## Abstract

In the title compound, C_4_H_5_N_3_O_2_, the nitro group is twisted with respect to the imidazole ring by a dihedral angle of 5.60 (2)°. Weak inter­molecular C—H⋯O and C—H⋯N hydrogen bonding is present in the crystal structure.

## Related literature

For the biological properties of nitro­imidazole derivatives, see: Hofmann (1953 ▶); Breccia *et al.* (1982 ▶); Boyer (1986 ▶). For their detonation properties, see: Storm *et al.* (1990 ▶); Katritzky *et al.* (1993 ▶); Bulusu *et al.* (1995 ▶). For the synthesis, see: Damavarapu *et al.* (2007 ▶).
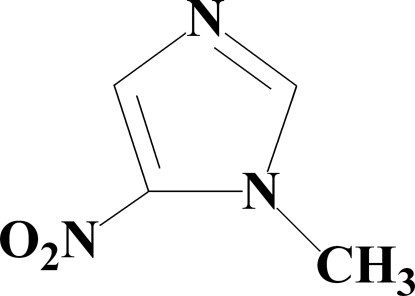


## Experimental

### 

#### Crystal data

C_4_H_5_N_3_O_2_
*M* *_r_* = 127.11
Orthorhombic, 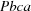

*a* = 5.323 (3) Å
*b* = 12.664 (6) Å
*c* = 15.993 (8) Å
*V* = 1078.1 (9) Å^3^
*Z* = 8
Mo *K*α radiation
μ = 0.13 mm^−1^
*T* = 113 K
0.30 × 0.26 × 0.10 mm

#### Data collection

Rigaku Saturn724 CCD diffractometer
Absorption correction: multi-scan (*CrystalClear*; Rigaku/MSC, 2005 ▶) *T*_min_ = 0.963, *T*_max_ = 0.987
10144 measured reflections
1272 independent reflections
1030 reflections with *I* > 2σ(*I*)
*R*_int_ = 0.059

#### Refinement

*R*[*F* ^2^ > 2σ(*F* ^2^)] = 0.036
*wR*(*F* ^2^) = 0.088
*S* = 1.01
1272 reflections
83 parameters
H-atom parameters constrained
Δρ_max_ = 0.17 e Å^−3^
Δρ_min_ = −0.32 e Å^−3^

### 

Data collection: *CrystalClear* (Rigaku/MSC, 2005 ▶); cell refinement: *CrystalClear*; data reduction: *CrystalClear*; program(s) used to solve structure: *SHELXTL* (Sheldrick, 2008 ▶); program(s) used to refine structure: *SHELXTL*; molecular graphics: *SHELXTL*; software used to prepare material for publication: *SHELXTL*.

## Supplementary Material

Crystal structure: contains datablock(s) I, global. DOI: 10.1107/S1600536811041705/xu5346sup1.cif

Structure factors: contains datablock(s) I. DOI: 10.1107/S1600536811041705/xu5346Isup2.hkl

Supplementary material file. DOI: 10.1107/S1600536811041705/xu5346Isup3.cml

Additional supplementary materials:  crystallographic information; 3D view; checkCIF report

## Figures and Tables

**Table 1:** Hydrogen-bond geometry (Å, °)

*D*—H⋯*A*	*D*—H	H⋯*A*	*D*⋯*A*	*D*—H⋯*A*
C2—H2⋯N1^i^	0.95	2.54	3.342 (2)	143
C4—H4*A*⋯O2^ii^	0.98	2.52	3.335 (2)	140
C4—H4*C*⋯O2^iii^	0.98	2.58	3.496 (2)	156

Symmetry codes: (i) 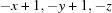; (ii) 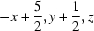; (iii) 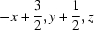.

**Fig. 1.**
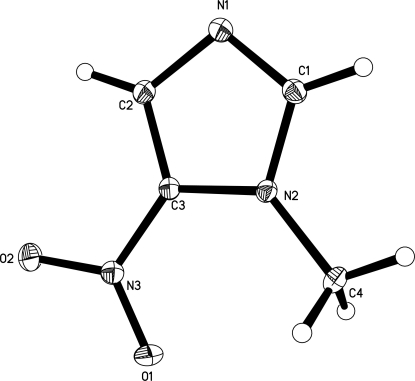
The molecular structure of the title compound with the atom numbering scheme. Displacement ellipsoids are drawn at the 30% probability level.

**Fig. 2.**
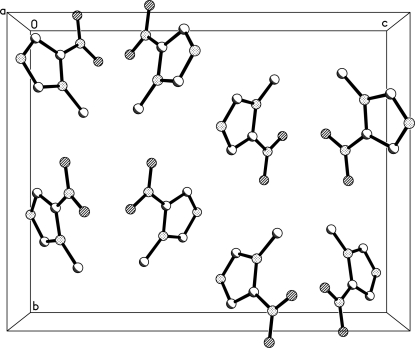
The crystal packing of the title compound.

## supplementary crystallographic information

### Comment

Nitroimidazole derivatives have been investigated extensively owing to their biological activity (Hofmann, 1953; Breccia *et al.*, 1982; Boyer, 1986). Recently, these so called "high energy density materials" have attracted renewed attention in conjunction with their favorable detonation performance (Storm *et al.*, 1990; Katritzky *et al.*, 1993; Bulusu *et al.*, 1995). 1-methyl-2,4,5-trinitroimidazole is a promising candidate, as a intermediate, 1-methyl-5-nitroimidazole was synthesized by the nitration of 1-methylimidazole (Damavarapu *et al.*, 2007). Here we report the crystal structure of the title compound (Fig. 1).

In the crystal structure, for the reason that the interaction of methyl group and nitro group, the nitro group is rotated out the imidazole plane, making dihedral angles of 5.60 (2)°.

### Experimental

The title compound was prepared according to literature method (Damavarapu *et al.*, 2007). Single crystals were obtained by evaporation of a solution of the title compound in dichloromethane at room temperature.

### Refinement

All H atoms were positioned geometrically and treated as riding, with C—H = 0.95 ° for imidazole ring H and 0.98 ° for methyl H atoms, and with U_iso_(H) = 1.2U_eq_(C) for imidazole ring H atom and 1.5U_eq_(C) for methyl H atoms.

### Crystal data

**Table:**

C_4_H_5_N_3_O_2_	*F*(000) = 528
*M**_r_* = 127.11	*D*_x_ = 1.566 Mg m^−^^3^
Orthorhombic, *P**b**c**a*	Mo *K*α radiation, λ = 0.71073 Å
Hall symbol: -P 2ac 2ab	Cell parameters from 3500 reflections
*a* = 5.323 (3) Å	θ = 1.6–27.8°
*b* = 12.664 (6) Å	µ = 0.13 mm^−^^1^
*c* = 15.993 (8) Å	*T* = 113 K
*V* = 1078.1 (9) Å^3^	Prism, colorless
*Z* = 8	0.30 × 0.26 × 0.10 mm

### Data collection

**Table:**

Rigaku Saturn724 CCD diffractometer	1272 independent reflections
Radiation source: rotating anode	1030 reflections with *I* > 2σ(*I*)
multilayer	*R*_int_ = 0.059
Detector resolution: 14.22 pixels mm^-1^	θ_max_ = 27.8°, θ_min_ = 2.6°
ω and φ scans	*h* = −6→6
Absorption correction: multi-scan (*CrystalClear*; Rigaku/MSC, 2005)	*k* = −16→16
*T*_min_ = 0.963, *T*_max_ = 0.987	*l* = −21→21
10144 measured reflections	

### Refinement

**Table:**

Refinement on *F*^2^	Primary atom site location: structure-invariant direct methods
Least-squares matrix: full	Secondary atom site location: difference Fourier map
*R*[*F*^2^ > 2σ(*F*^2^)] = 0.036	Hydrogen site location: difference Fourier map
*wR*(*F*^2^) = 0.088	H-atom parameters constrained
*S* = 1.01	*w* = 1/[σ^2^(*F*_o_^2^) + (0.0505*P*)^2^] where *P* = (*F*_o_^2^ + 2*F*_c_^2^)/3
1272 reflections	(Δ/σ)_max_ < 0.001
83 parameters	Δρ_max_ = 0.17 e Å^−^^3^
0 restraints	Δρ_min_ = −0.32 e Å^−^^3^

### Special details

**Table:**

Geometry. All e.s.d.'s (except the e.s.d. in the dihedral angle between two l.s. planes) are estimated using the full covariance matrix. The cell e.s.d.'s are taken into account individually in the estimation of e.s.d.'s in distances, angles and torsion angles; correlations between e.s.d.'s in cell parameters are only used when they are defined by crystal symmetry. An approximate (isotropic) treatment of cell e.s.d.'s is used for estimating e.s.d.'s involving l.s. planes.
Refinement. Refinement of *F*^2^ against ALL reflections. The weighted *R*-factor *wR* and goodness of fit *S* are based on *F*^2^, conventional *R*-factors *R* are based on *F*, with *F* set to zero for negative *F*^2^. The threshold expression of *F*^2^ > σ(*F*^2^) is used only for calculating *R*-factors(gt) *etc*. and is not relevant to the choice of reflections for refinement. *R*-factors based on *F*^2^ are statistically about twice as large as those based on *F*, and *R*- factors based on ALL data will be even larger.

### Fractional atomic coordinates and isotropic or equivalent isotropic displacement parameters (Å^2^)

**Table:**

	*x*	*y*	*z*	*U*_iso_*/*U*_eq_	
O1	1.21610 (15)	0.62227 (7)	0.20073 (5)	0.0222 (2)	
O2	1.10195 (15)	0.47117 (7)	0.14851 (6)	0.0272 (3)	
N1	0.54173 (19)	0.64301 (8)	0.03187 (6)	0.0201 (3)	
N2	0.80463 (17)	0.72006 (7)	0.12271 (6)	0.0150 (2)	
N3	1.07644 (17)	0.56758 (8)	0.15745 (6)	0.0175 (2)	
C1	0.6053 (2)	0.73142 (10)	0.07171 (7)	0.0179 (3)	
H1	0.5183	0.7963	0.0648	0.021*	
C2	0.7109 (2)	0.56985 (9)	0.05864 (7)	0.0184 (3)	
H2	0.7152	0.4980	0.0416	0.022*	
C3	0.87376 (19)	0.61580 (9)	0.11396 (7)	0.0154 (3)	
C4	0.9175 (2)	0.80490 (9)	0.17239 (7)	0.0194 (3)	
H4A	1.0842	0.8217	0.1501	0.029*	
H4B	0.9332	0.7819	0.2307	0.029*	
H4C	0.8105	0.8678	0.1696	0.029*	

### Atomic displacement parameters (Å^2^)

**Table:**

	*U*^11^	*U*^22^	*U*^33^	*U*^12^	*U*^13^	*U*^23^
O1	0.0187 (5)	0.0260 (5)	0.0220 (5)	−0.0025 (4)	−0.0049 (3)	−0.0034 (4)
O2	0.0288 (5)	0.0155 (5)	0.0373 (6)	0.0058 (4)	−0.0066 (4)	−0.0020 (4)
N1	0.0223 (6)	0.0185 (6)	0.0196 (5)	−0.0001 (4)	−0.0043 (4)	0.0009 (4)
N2	0.0164 (5)	0.0132 (5)	0.0153 (5)	−0.0020 (4)	−0.0003 (4)	0.0010 (4)
N3	0.0170 (5)	0.0176 (5)	0.0179 (5)	0.0002 (4)	0.0003 (4)	0.0000 (4)
C1	0.0165 (6)	0.0183 (6)	0.0188 (6)	0.0003 (5)	0.0000 (4)	0.0028 (5)
C2	0.0208 (6)	0.0157 (6)	0.0186 (6)	−0.0012 (5)	−0.0016 (5)	−0.0007 (5)
C3	0.0159 (6)	0.0142 (6)	0.0161 (5)	0.0002 (4)	0.0002 (4)	0.0006 (4)
C4	0.0217 (6)	0.0150 (6)	0.0215 (6)	−0.0037 (5)	−0.0003 (5)	−0.0028 (5)

### Geometric parameters (Å, °)

**Table:**

O1—N3	1.2294 (12)	N3—C3	1.4215 (14)
O2—N3	1.2368 (14)	C1—H1	0.9500
N1—C1	1.3319 (16)	C2—C3	1.3687 (16)
N1—C2	1.3610 (15)	C2—H2	0.9500
N2—C1	1.3459 (15)	C4—H4A	0.9800
N2—C3	1.3778 (16)	C4—H4B	0.9800
N2—C4	1.4651 (15)	C4—H4C	0.9800
			
C1—N1—C2	104.69 (10)	N1—C2—H2	125.3
C1—N2—C3	104.56 (9)	C3—C2—H2	125.3
C1—N2—C4	124.99 (10)	C2—C3—N2	107.69 (10)
C3—N2—C4	130.42 (10)	C2—C3—N3	127.92 (11)
O1—N3—O2	123.70 (10)	N2—C3—N3	124.38 (10)
O1—N3—C3	119.50 (10)	N2—C4—H4A	109.5
O2—N3—C3	116.80 (10)	N2—C4—H4B	109.5
N1—C1—N2	113.60 (10)	H4A—C4—H4B	109.5
N1—C1—H1	123.2	N2—C4—H4C	109.5
N2—C1—H1	123.2	H4A—C4—H4C	109.5
N1—C2—C3	109.45 (11)	H4B—C4—H4C	109.5
			
C2—N1—C1—N2	−0.13 (13)	C4—N2—C3—C2	−178.47 (10)
C3—N2—C1—N1	0.39 (13)	C1—N2—C3—N3	−179.24 (10)
C4—N2—C1—N1	178.52 (10)	C4—N2—C3—N3	2.77 (18)
C1—N1—C2—C3	−0.19 (12)	O1—N3—C3—C2	174.93 (10)
N1—C2—C3—N2	0.43 (13)	O2—N3—C3—C2	−4.59 (17)
N1—C2—C3—N3	179.13 (10)	O1—N3—C3—N2	−6.56 (16)
C1—N2—C3—C2	−0.48 (12)	O2—N3—C3—N2	173.92 (10)

### Hydrogen-bond geometry (Å, °)

**Table:**

*D*—H···*A*	*D*—H	H···*A*	*D*···*A*	*D*—H···*A*
C2—H2···N1^i^	0.95	2.54	3.342 (2)	143
C4—H4A···O2^ii^	0.98	2.52	3.335 (2)	140
C4—H4C···O2^iii^	0.98	2.58	3.496 (2)	156

Symmetry codes: (i) −*x*+1, −*y*+1, −*z*; (ii) −*x*+5/2, *y*+1/2, *z*; (iii) −*x*+3/2, *y*+1/2, *z*.
